# Multilingual end-to-end ASR for low-resource Turkic languages with common alphabets

**DOI:** 10.1038/s41598-024-64848-1

**Published:** 2024-06-15

**Authors:** Akbayan Bekarystankyzy, Orken Mamyrbayev, Mateus Mendes, Anar Fazylzhanova, Muhammad Assam

**Affiliations:** 1 Satbayev University, Almaty, Kazakhstan; 2 Narxoz University, Almaty, Kazakhstan; 3 Institute of Information and Computational Technologies, Almaty, Kazakhstan; 4 Polytechnic Institute of Coimbra, ISEC, Coimbra, Portugal; 5 University of Coimbra, ISR, Coimbra, Portugal; 6 Committee of Science of the Ministry of Science and Higher Education of the RK, Institute of Linguistics and Named After Akhmet Baitursynuly, Almaty, Kazakhstan; 7 University of Science and Technology, Bannu, KP Pakistan

**Keywords:** End-to-end ASR, Agglutinative languages, Multilingual learning, Conformer, Attention-based, Connectionist temporal classification, Low-resource languages, Computer science, Information technology, Scientific data

## Abstract

To obtain a reliable and accurate automatic speech recognition (ASR) machine learning model, it is necessary to have sufficient audio data transcribed, for training. Many languages in the world, especially the agglutinative languages of the Turkic family, suffer from a lack of this type of data. Many studies have been conducted in order to obtain better models for low-resource languages, using different approaches. The most popular approaches include multilingual training and transfer learning. In this study, we combined five agglutinative languages from the Turkic family—Kazakh, Bashkir, Kyrgyz, Sakha, and Tatar,—in order to provide multilingual training using connectionist temporal classification and an attention mechanism including a language model, because these languages have cognate words, sentence formation rules, and alphabet (Cyrillic). Data from the open-source database Common voice was used for the study, to make the experiments reproducible. The results of the experiments showed that multilingual training could improve ASR performances for all languages included in the experiment, except Bashkir language. A dramatic result was achieved for the Kyrgyz language: word error rate decreased to nearly one-fifth and character error rate decreased to one-fourth, which proves that this approach can be helpful for critically low-resource languages.

## Introduction

Kazakh language is a low-resource language from the Turkic family of languages, which belong to agglutinative languages. Almost all of these languages suffer from data shortages^1^. Namely, they suffer from a lack of transcribed audio data. For example, the largest open-source corpus, which is an Open-Source Uzbek Speech Corpus^2^, has only 105 h of audio transcribed. There were 258 recorded hours and 97 validated hours in Uzbek in Common Voice. Common Voice is a Mozilla’s project for collecting open-source datasets of transcribed audio data for all possible languages in the world. Anyone can participate in improving this resource. ISAAI’s Kazakh language corpus contains 335 h^3^ of transcribed audio (2 h in Common Voice). Some agglutinative languages only have corpuses in the Common Voice. 

Inspired by the results presented in some papers^4,5^, we decided to try a multilingual model using datasets of several agglutinative languages because multilingual models demonstrate stable gains over monolingual models^4^. Therefore, it is supposed that the multilingual model taken for the group of agglutinative languages can decrease character error rate (CER) and word error rate (WER) for distinct languages included in the experiments. Moreover, it is assumed that the model can be used as a basis for providing transfer learning for distinct groups of languages.

Experiments provided on 11 languages from the Roman family give better results on multilingual deep neural networks (DNN) compared to monolinguals^6^. Transfer learning conducted using the English language model significantly improved the CER for 12 languages (some of them agglutinative languages)^5^. The reason for the hypothesis of the current study is that Turkic languages have common characteristics including linguistic structure, agglutinative morphology, and vowel harmony^7^. Furthermore, attempts to use a language model of the Russian language to Kazakh^8^ resulted in unsatisfactory Error Rates because word formations of these languages are different^1^.

According to previous research, it was decided to train a multilingual model for languages from the Turkic family using the Cyrillic alphabet. The reason for choosing such a method is that the similarity of the alphabet can lead to better compatibility among language words that have common roots.

Some of the existing combining methods showed large improvements on the datasets of related languages. Among them it is possible to mention the study^9^, with unsupervised cross-lingual training for speech recognition. In this study the authors detected that the pretrained model on the collection of datasets, implicitly trained to classify related languages, also has shown that low-resource languages can have benefits from training over related languages, rather than unrelated languages. Moreover, authors of the paper about multilingual training using the datasets from GlobalPhone^10^ mentioned that the use of phonetically related languages has more benefits over the use of unrelated languages, while one more study^11^ showed that positive impact can be achieved even in the case of training unrelated languages which have similarities in phonology.

But none of the mentioned studies assess the impact of script similarity of related languages. The basic idea of this research is to study the impact of combining languages from the Turkic language family, with similar scripts. Common word and sentence formation rules of the selected languages with similar scripts allow to get a working model for each of the languages included in the experiments. The contributions of this research are:

1. Development of an ASR method for critically low-resource languages;
2. Improvement of ASR performance for languages from one language family (Turkic).

"Related work" section presents a review of relevant work found in the literature. "Results" section describes the datasets and the methods used in this study. "Materials and methods" section presents the main results of the study. "Discussion" section contains a discussion of the results. "Conclusion" section  draws conclusions and proposes directions for future work.

## Related work

Pooling resources from different languages is helpful for low-resource languages^10,12–15^. The same idea can also be applied to distinct language families. Investigations on combining experiments, such as Transfer and Multilingual training on the languages of Turkic family, and improvements in WER and CER, were mentioned in^1,16,17^. Applications of ESPNet and its benefits for languages of this family can be found in^2,13^. ESPNet is a deep neural network-based automatic speech recognition toolkit that was proposed in 2018^18^. ESPNet’s conformer encoder and transformer decoder, which are used for low-resource languages, obtained an improvement of more than 15%^19,20^. The application of the conformer encoder in the multilingual end-to-end (E2E) model yielded better results than the others^13^.

Suggestions for further use of multilingual models as a basis for transfer learning could be justified by the results of^21^. A multilingual deep neural network (DNN) and a matrix factorization algorithm were used to extract high-level features. In the second stage, the authors applied a joint CTC-attention mechanism with shallow recurrent neural networks (RNNs) for high-level features extracted from previous levels. The authors state that the proposed architecture is the best among all the existing end-to-end transfer models. The use of a multilingual model in^22^, built on a collection of adversarial languages for further providing transfer learning for absolutely different languages, shows improvement by decreasing WER up to 10.1%. This study uses the IARPA Babel. IARPA Babel is a program aimed at improving automatic speech recognition in a large number of different languages. Using sub-word units in a CTC-attention-based system on the LibriSpeech 1000 h dataset obtained an improvement over a character-based hybrid system, reducing the WER to 12.8% without a language model^23^. Moreover, the authors stated that using sub-words helps to avoid out-of-vocabulary problems. This can be assessed as a reason for choosing languages from one language family because a large part of the words from these languages have common roots.

Another successful example of using end-to-end ASR for agglutinative languages can be found in^24^. In this study, experiments were conducted using a conformer model and CTC-Attention on data augmentation, noise injection, and exponential moving average. For the Corpus of Spontaneous Japanese, the authors achieved a state-of-the-art CER of 3.2%. The Introduction contains a summary of the information about previous works on Turkic languages and the two previous paragraphs of this section show the reasonability of the models and network chosen for the experiments in this study.

Experiments in^5^ were conducted using Mozilla’s Deep Speech v0.3.0^25^. A total of 26 h of data for Tatar and 10 h of data for Turkish were used to train the ASR model for these languages over the English language model. The CER for Tatar was 26.42%, whereas for Turkish it was 27.52%.

A deep neural network with hidden Markov model (DNN-HMM) system was applied to Turkish, one of the most popular and widely used Turkic languages. The dataset consisted of 6.1 h of data, collected from mobile devices. In comparison with GMM-HMM systems, the authors obtained a WER of approximately 2.5 in comparison with the GMM-HMM systems.

The authors of^26^ investigated questions of speech recognition in emergency call centres for the Azerbaijani language. In the experiments, two types of datasets were used: dialogue dataset (27 h) and summary dataset (57 h). The GMM-HMM and DNN-HMM were applied to train the acoustic model. The authors found that the DNN-HMM showed better results in the experiments, and the trigram language model gave no risk of overfitting.

In^27^, the authors considered a transformer architecture with self-attention components that can shorten the training process by parallelizing the processes for the recognition of speech in the Kazakh language. Application of the Transformer + CTC LM model decreased the CER and WER to 3.7% and 8.3%, respectively, for 200 h of read speech.

The hybrid model used in^21^ comprised a CTC with an attention mechanism for 400 h of data. The results were as follows: CER = 9.8% and WER = 15.3%. Including Language Model (LM) in this composition led to a significant decrease in the CER and WER (5.8%, 12.2%).

The end-to-end conformer model in^2^ for the Uzbek language for 105 h of data volume gave more effective results over E2E + LSTM and E2E + Transformer for Uzbek language from the Turkic family. The end-to-end conformer model showed lower error rates (CER = 5.8%, WER = 17.4%) when the Language Model was included in the decoder.

In^1^, an attempt was made to fit a model trained on the Kazakh language dataset to the Azaerbaijani dataset. In this experiment, the (NMF) algorithm was used to extract features from the audio data. NMF is necessary to reduce hidden level outputs and decrease redundant values from high-level vectors^21^. Furthermore, these characteristics were trained on the attention mechanism of the joint CTC. This approach gave Phoneme Error Rate (PER) equal to 14.23%.

Authors of^16^ study single E2E automatic speech recognition (ASR) using the ESPNet toolkit for the commonly used languages in Kazakhstan: Kazakh (KZ), English (EN), and Russian (RU). The combined dataset of the three languages has a total volume of 975.6 h. To solve the issue of grapheme compatibility, the authors also combined the grapheme sets of all languages. The training results showed an average WER of 20.5%.

Authors of^17^ provided a study on multilingual training for the languages of Turkic family including Azerbaijani, Bashkir, Chuvash, Kyrgyz, Sakha, Tatar and Uyghur languages. Experiments in this work were also provided by the use of a conformer architecture changing values of hyperparameters. The main difference of the mentioned study from the current paper is the fact that languages have different scripts and belong to different branches of Turkic languages.

Table 1 shows a summary of the comparative results of different models for different volumes of data for the Kazakh and all mentioned Turkic languages. The table compares the CER and WER achieved using the different models and dataset sizes.

**Table 1:** Summary of different models and approaches applied for agglutinative languages.

Model	Language	CER (%)	WER (%)	Volume of data (hours)
CTC and attention^27^	Kazakh	9.8	15.3	400
CTC and attention + LM^27^	Kazakh	5.8	12.2	400
Transformer and CTC + LM^27^	Kazakh (Read speech)	3.7	8.3	200
Transformer and CTC + LM^27^	Kazakh (Conversational telephone speech)	9.6	15.8	200
E2E-Conformer^2^	Uzbek	7.5	21.2	105
E2E-Conformer + LM^2^	Uzbek	5.8	17.4	105
Transformer architecture^16^	Combined data of Kazakh, English and Russian languages	n.a	20.5	975.6
Transfer learning over English ASR model on DeepSpeech (A six-layer unidirectional CTC model, with one LSTM layer)^5^	Tatar	26.42	n.a	26
Transfer learning over English ASR model on DeepSpeech (A six-layer unidirectional CTC model, with one LSTM layer)^5^	Turkish	27.55	n.a	10
Conformer with CVC receipe for all Turkic languages^17^	Azerbaijan	26.7	75.9	0.13
Conformer with CVC receipe for all Turkic languages^17^	Bashkir	1.5	4.9	232.37
Conformer with CVC receipe for all Turkic languages^17^	Chuvash	4.9	17.2	11.9
Conformer with CVC receipe for all Turkic languages^17^	Kyrgyz	4.9	13.1	18.58
Conformer with CVC receipe for all Turkic languages^17^	Sakha	15.7	45	6.61
Conformer with CVC receipe for all Turkic languages^17^	Tatar	5.6	18.1	25.07
Conformer with CVC receipe for all Turkic languages^17^	Uyghur	4.1	11.0	35.61
Conformer with CVC receipe for all Turkic languages^17^	Kazakh	11.7	29	Common Voice Corpus 10.0: 1.60 Kazakh Speech Corpus: 332.6
Conformer with CVC receipe for all Turkic languages^17^	Turkish	3.3	9.0	Common Voice Corpus 10.0: 51.46 Turkish Speech Corpus:218.24
Conformer with CVC receipe for all Turkic languages^17^	Uzbek	3.0	10.3	Common Voice Corpus 10.0: 94.24 Uzbek Speech Corpus: 104.90

## Materials and methods

### Datasets

Almost all Turkic languages have common rules of word formation, and words can have the same meaning in these languages. Table 2 shows some examples of phrases formed using words with similar soundings and meanings for the Kazakh, Kyrgyz and Tatar languages, which belong to the Turkic language family and are included in the study.

**Table 2:** Comparative table of expressions with the same meaning in Kazakh, Kyrgyz and Tatar languages.

Kazakh	Kyrgyz	Tatar	English meaning
бip aлмa [biyr alma]	биp aлмa [bir alma]	бep aлмa [ber alma]	One apple
тepeң көл [tereng kyol]	тepeң көл [tereng kyol]	тиpән күл [tiraen qul]	Deep lake
aқ дoп [aaq dop]	aк тoп [aaq top]	aк тyп [aack tup]	White ball
қapa қoй [qara qoy]	кapa кoй [kara koy]	кapa кyй [qara quy]	Black sheep

To make the experiment reproducible, data from the open-source dataset Common Voice was used in the present work. Another reason for choosing this open-source resource is that the dataset for the Kazakh language is only one hour long. This allows to observe the effect of a multilingual approach on critically low-resource languages. When the experiments of the current investigation began, Mozilla’s Common Voice Corpus 8.0^5^ was the latest available.

Languages with Cyrillic scripts were chosen from the dataset: Kazakh (1 h), Bashkir (265 h), Kyrgyz (44 h), Tatar (29 h), and Sakha (6 h). The datasets mentioned contain a wide range of speaker ages for both males and females, as shown in Tables 3 and 4. For some languages, there are no samples of older speakers, and in the case of Tatar, there are no samples of speakers less than 19 years of age. Male speakers were predominant in terms of gender, with the exception of Bashkir.

**Table 3:** Distribution of the data used–number of speakers, by age.

Languages	Total hours (validated)	Ages (%)					
		< 19	19–29	30–39	40–49	50–59	60–69
Tatar	29		5	73		1	
Kazakh	1	6	26	3		11	
Sakha (Yakut)	6	11	2	44	7		
Bashkir	265 (255)	4	17	17	6	5	20
Kyrgyz	6.5	19	67	8	1

**Table 4:** Distribution of the data used–number of speakers, by gender.

Languages	Gender	
	Male	Female
Tatar	79	2
Kazakh	42	3
Sakha (Yakut)	54	10
Bashkir	30	40
Kyrgyz	54	36

### Speech recognition models

The ESPnet^18^ was choosen as a toolkit for training multilingual and monolingual ASR systems in the study. ESPnet supports conformer architecture^20^, which was successfully applied for languages of Turkic family^16,17^. The initial architecture for this experiment was taken from^16^, as in this study authors could get promising results for multilingual training over Kazakh, Russian and English languages.

The experiments included five agglutinative languages with Cyrillic scripts: Bashkir (ba), Kazakh (kk), Kyrgyz (ky), Sakha (sah), and Tatar (tt), with the next grapheme set $\left({Gr}_{ba},{Gr}_{kk},{Gr}_{ky,}{Gr}_{sah},{Gr}_{tt}\right)$. The grapheme set is understood using letters of the languages present in our experiment. Examples of grapheme letters in the five languages are listed in Table 5. Bold and red letters indicate specific letters for distinct languages.

**Table 5:** Graphemes for Turkic languages with Cyrillic alphabet, included in the experiments.

ba	ky	sah	kk	tt	All
a	a	a	a	a	a
ә			ә	ә	ә
б	б	б	б	б	б
в	в	в		в	в
г	г	г	г	г	г
ғ			ғ		ғ
		ҕ			ҕ
д	д	д	д	д	д
e	e	e	e	e	e
ё	ё			ё	ё
ж	ж	ж	ж	ж	ж
				җ	җ
ҙ					ҙ
з	з	з	з	з	з
и	и	и	и	и	и
			i		i
й	й	й	й	й	й
к	к	к	к	к	к
ҡ					ҡ
			қ		қ
л	л	л	л	л	л
м	м	м	м	м	м
н	н	н	н	н	н
	ӊ				ӊ
		ҥ			ҥ
ң	ң		ң	ң	ң
o	o	o	o	o	o
ө	ө	ө	ө	ө	ө
п	п	п	п	п	п
p	p	p	p	p	p
c	c	c	c	c	c
ҫ					ҫ
т	т	т	т	т	т
y	y	y	y	y	y
ү	ү	ү	ү	ү	ү
			ұ		ұ
ф	ф	ф		ф	ф
x	x	x	x	x	x
h		h		h	h
ц	ц	ц		ц	ц
ч	ч	ч		ч	ч
ш	ш	ш	ш	ш	ш
щ	щ	щ	щ	щ	щ
	ъ			ъ	ъ
	ы	ы	ы	ы	ы
	ь	ь		ь	ь
	э	э		э	э
	ю	ю	ю	ю	ю
		я	я	я	я

The training sets for each language are defined as a pair $\left\{{X}_{i},{Y}_{i}\right\}$ for language $i$. Therefore, the datasets are defined as follows (1):

1 $$\left\{{X}_{i},{Y}_{i}:i\in \left(ba,kk,ky,tt,sah\right)\right\}$$

where ${X}_{i}$ is an input given as acoustic features, ${Y}_{i}$ is the corresponding output or target sequence of characters. The training and grapheme datasets for the multilingual language model were combined from the data for distinct languages according to^16^.

The multilingual dataset is defined as the union of the five datasets of the languages.

2 $$\left\{{X}_{all},{Y}_{all}\right\}=\left\{{X}_{ba},{Y}_{ba}\right\}\cup \left\{{X}_{kk},{Y}_{kk}\right\}\cup \left\{{X}_{ky},{Y}_{ky}\right\}\cup \left\{{X}_{tt},{Y}_{tt}\right\}\cup \left\{{X}_{sah},{Y}_{sah}\right\}$$

The multilingual grapheme set is the union of the five grapheme sets present in the datasets included in the experiment:

3 $${Gr}_{all}={Gr}_{ba}\cup {Gr}_{kk}\cup {Gr}_{ky}\cup {Gr}_{tt}\cup {Gr}_{sah}$$

**Figure 1:**
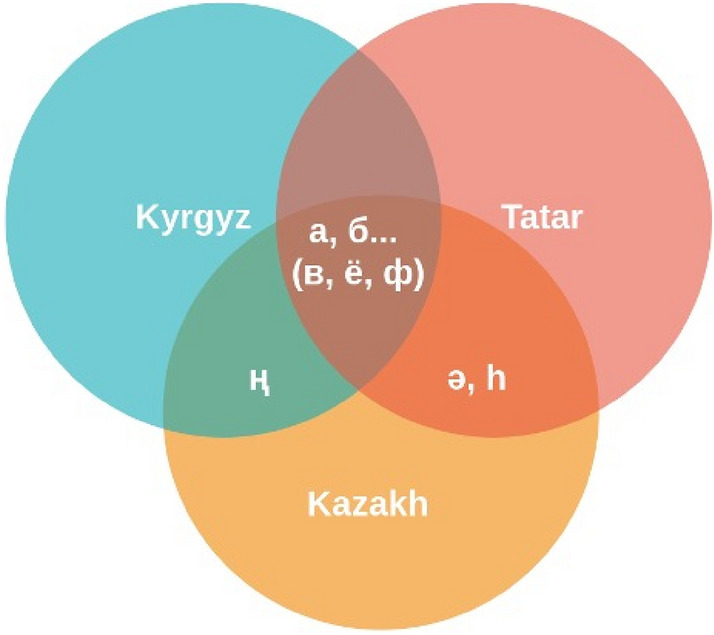
Example of common letters for some of the languages included in the experiments.

For languages with critically low-transcribed data, it is possible to miss some letters and sounds in the dataset. The chosen approach of combining the data of the languages with common scripts and language families can close the gap of absence. For example, in Table 4, formed on the basis of letters from the datasets, letters ‘в’ [vae], ‘ё’ [io], and ‘ф’ [fae] are absent for the Kazakh language. However, these letters exist in the Kazakh alphabet and are often used in words from other languages. In addition, some examples of common letters for all languages included in the experiment (Bashkir, Kazakh, Kyrgyz, Sakha, and Tatar) and common letters for only some of them (e.g., between Tatar and Kazakh) are presented in Fig. 1. It is important to note that the soundings of these letters are similar in all these languages. This implies that the proposed multilingual approach can help improve the ASR model for critically low-resource languages.

For training, all E2E ASR models used conformer encoder and transformer decoders. Connectionist Temporal Classification (CTC) and attention mechanisms were used in both stages of training: encoding and decoding. The weights of CTC and Attention in the hybrid model were given by the hyper-parameter ctc_weight_. This parameter was left in its default value: ${\gamma }_{CTC}={ctc}_{weight}=0.3$, because in^6^ it was proved that this proportion is the best among other values. The weight of the attention mechanism is ${\gamma }_{att}=0.7$ according to (4).

4 $${\gamma }_{CTC}=1-{\gamma }_{att}$$

where $\gamma$ is the coefficient that controls the weights of the CTC and attention mechanism^4^. This coefficient is also used in decoding, considering the weights of the model (5):

5 $$log{p}_{hyp}=\gamma log{p}_{CTC}+\left(1-\gamma \right)lop{p}_{att}$$

In (5), ${p}_{hyp}$ is a score used in the beam search^4^. Probabilities are applicable to each output character.

**Figure 2:**
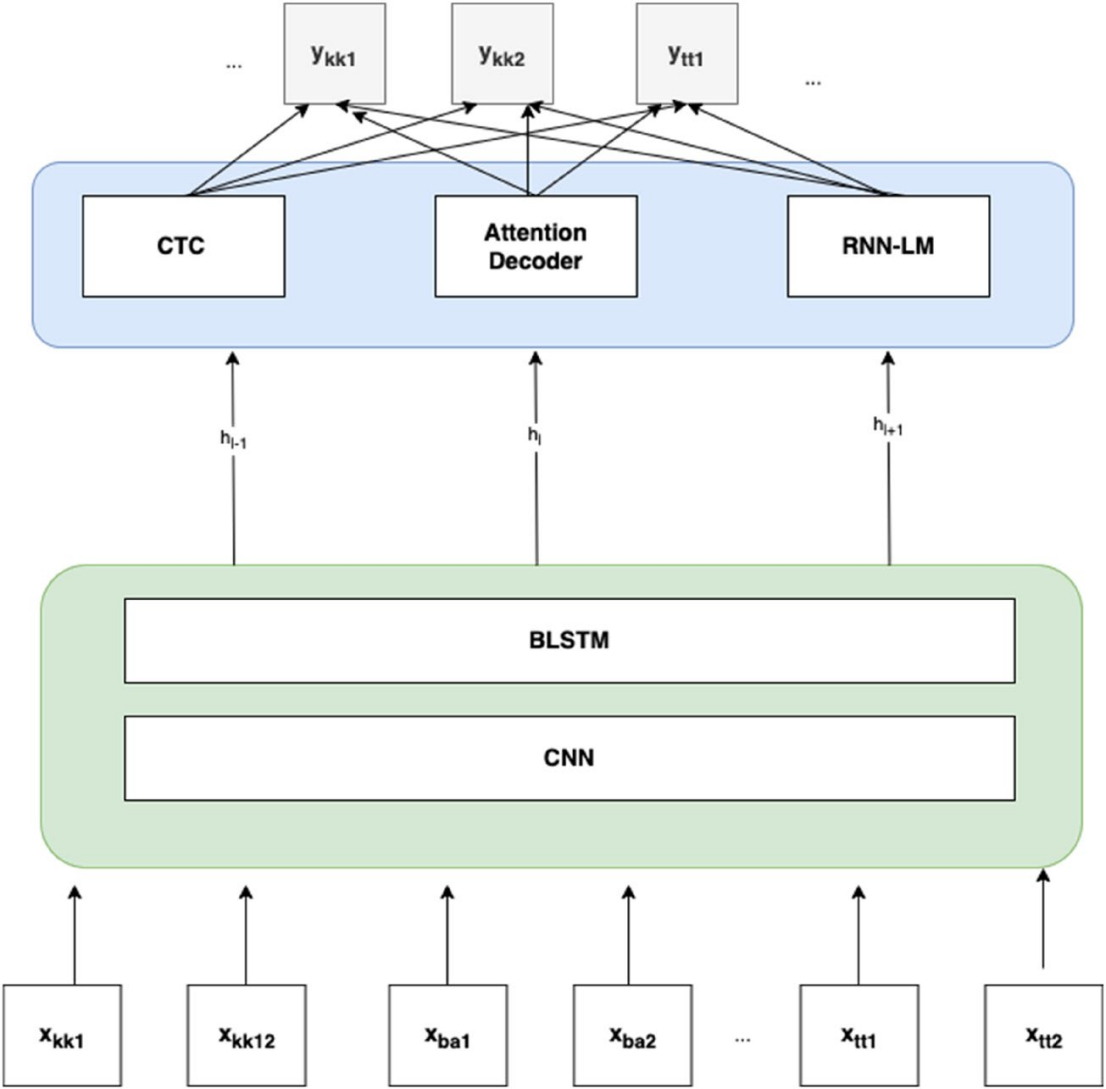
Network architecture for multilingual model of Turkic languages with Cyrillic alphabet.

Figure 2 shows the architecture of the system used for training the multilingual model The conformer architecture has an encoder with CNN and BLSTM layers with 12 blocks and 4 attention heads, followed by a joint decoder which consists of three components: namely CTC, Attention decoder and RNN LM. The transformer decoder with 6 blocks, which have 2048 linear units, receives hidden layer data from the encoder. The weight of CTC in decoding is 0.3, the same weight has RNN LM. Feature extraction type is set by feats_type parameter. This parameter was set to fbank_pitch, because in^15^ it was found that features extracted by applying filter bank and pitch methods in training CTC decreased the CER value. The activation function used is swish, the optimizer is adam. Learning rate was chosen as 4, due to the critically low size of the data.

In this study, a deep Convolutional Neural Network (CNN) was chosen as an encoder function.

Here the method of combining the input features according to formulas (2) and (3) was used. The output sequence was tested for each language distinctly.

### Code availability

The code for training the multilingual ASR system can be found by the link https://github.com/akbayanb/espnetMultilingualCyrillic/tree/master/egs2/commonvoice/asr1_all.

## Results

### Monolingual ASR models

The first monolingual ASR models were trained using the training datasets $\left\{{{\varvec{X}}}_{{\varvec{i}}},{{\varvec{Y}}}_{{\varvec{i}}}\right\}$ for each distinct language, where ${\varvec{i}}\in \{{\varvec{k}}{\varvec{k}},{\varvec{b}}{\varvec{a}},{\varvec{k}}{\varvec{y}},{\varvec{s}}{\varvec{a}}{\varvec{h}},{\varvec{t}}{\varvec{t}}$}. Speed perturbation was applied to languages with critically low-resource data. Only the Bashkir language was trained without speed perturbation. The CER and WER results obtained for the validation and test sets are listed in Table 6. In the initial training, the results for the Kyrgyz language showed that the CER and WER were lower in the test set than in the training set. Considering this error, duplicates from the Kyrgyz corpus were removed, and only 6.5 h of data were kept from the initial 44 h. In Table 6 for Bashkir language 265 is the total amount of data, 255 is the verified amount of data in hours.

**Table 6:** Monolingual ASR model results.

Languages	Details	Total hours	# Uttr-s	Validation set		Test set	
				CER	WER	CER	WER
Tatar	s.p.(speed perturbation): 0.9,1.0,1.1	29	train: 20,204, val: 2812	4.5	17.0	7.0	22.5
Kazakh	s.p.: 0.9,1.0,1.1	1	train: 406, val: 316	66.3	123.6	67.7	124.2
Sakha (Yakut)	s.p.: 0.9,1.0,1.1	6	train: 1633, val: 1083	29.2	79.7	32.8	85.5
Bashkir	no s.p	265 (255)	train: 178,522, val: 14,577	1.8	6.4	1.7	6.1
Kyrgyz	s.p.: 0.9,1.0,1.1	44(6.5 kept)	train: 4010, val: 502	17.7	54.3	17.9	55.3

### Multilingual ASR models

The multilingual ASR model was trained on the basis of the multilingual dataset $\left\{{X}_{all},{Y}_{all}\right\}$ with the following overall utterances: train, 227,031; test, 20,401. Speed perturbation was not applied to the multilingual ASR model. Test folders of the distinct languages were used for decoding. Comparing data from Tables 6 and 7, especially test/WER and test/CER, it is possible to conclude that multilingual ASR gives very promising results for languages with critically low-resource data: test/WER for Kazakh language decreases from 124.2 to 64.3, test/CER decreases from 67.7 to 19.3. For Kyrgyz language with 6.5 h of data, multilingual model’s CER decreased from 17.9 to 3.9 and WER decreased from 55.3 to 11.4 for the data from the test set. The improvement for Kyrgyz language in the experiment is the best case in the current study. For Tatar language, the decreases in error rates are not significant, which proves the fact that this type of multilingual training is applicable only to critically low resource languages. The results for Sakha language are smaller in comparison with others, because other languages (Bashkir, Kazakh, Kyrgyz and Tatar) belong to Kipchak branch Turkic family while Sakha belongs to Siberian Turkic branch. Character Error Rate for the test set of Sakha language decreased from 32.8 to 18, while Word Error Rate changed from 85.5 to 58..

**Table 7:** Multilingual ASR model results.

Languages	Validation set		Test set	
	CER	WER	CER	WER
ALL: Cyrillic	3.8	11.8		
Tatar	n.a	n.a	5.3	19.7
Kazakh	n.a	n.a	19.3	64.3
Sakha (Yakut)	n.a	n.a	18.0	58.0
Bashkir	n.a	n.a	1.7	6.4
Kyrgyz	n.a	n.a	3.9	11.4

**Figure 3:**
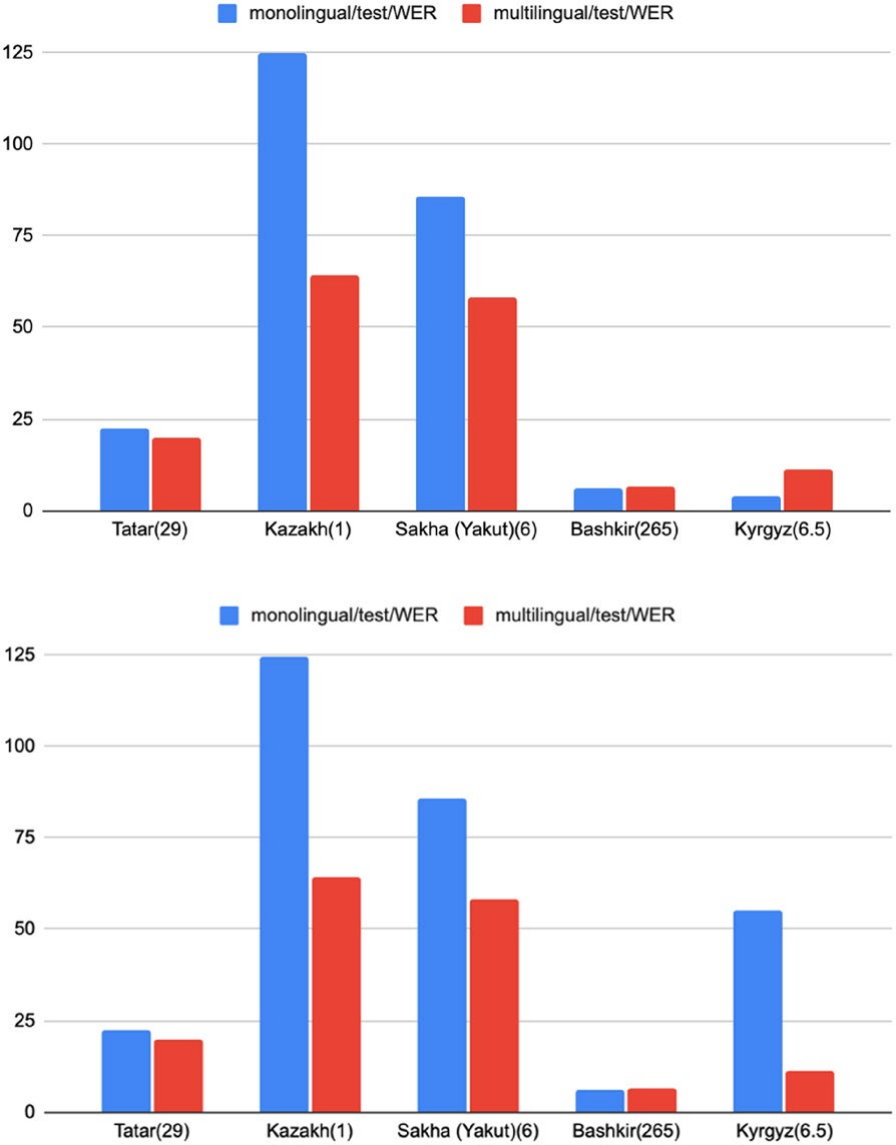
Monolingual ASR model WER comparison with multilingual training.

A comparison graph based on Tables 6 and 7 for test/WER is presented in Fig. 3. Here, it is possible to determine that for the language with maximum hours, Bashkir’s language, the results of multilingual and monolingual models are nearly the same. Kyrgyz, Sakha and Tatar have also made some improvements.

## Discussion

The results obtained in this study show significant differences in comparison with the results of^5^. The model and data used in^5^ are different, although there are some similarities in the approach followed. Transfer learning results for languages from the Turkic family over the English language model, especially for the Tatar language, showed a higher CER than in our investigation (26.42%). In this case, the difference in the amount of data (authors of^5^ trained with 26 h of Tatar language speech, while in the current experiment were used 29 h of Tatar speech) must be taken into account. In our experiments, the CER was lower in the multilingual model (5.3) than in the monolingual model (7.0) for Tatar.

The results of^4^, obtained by training languages from different language groups, provided improvements for all languages included in the experiment. But there is no dramatic improvement as in the current study: in our investigation, ASR gives very promising results for languages with critically low-resource data: test set WER for Kazakh language decreases from 124.2 to 64.3, test set CER decreases from 67.7 to 19.3.

As Kazakh and Kyrgyz languages have more similarities with each other, they have more improvements from multilingual training in comparison with Sakha language. For 6.5 h of Kyrgyz language, in the test set CER decreased from 17.9 to 3.9 and WER decreased from 55.3 to 11.4. Results for Kazakh language are given in the previous paragraph. For Tatar language, error rates decreased not significantly, which confirms our suggestion that multilingual training for languages with common scripts is applicable only to critically low resource languages. Results for Bashkir language have minor changes, as this language has sufficient amount of data. The results for Sakha are not as good as for Kyrgyz language, because this language is different in comparison with other languages included in the experiment. But the similarity of scripts and total amount of collected data could serve as a reason for the decrease in CER from 32.8 to 18.0, and in WER from 85.5 to 58.0 for test set.

The results of this work prove that multilingual training with CTC + Attention mechanisms, including language models, can help obtain meaningful results for languages with critically low-level data. But the advantage of the approach is its drawback at the same time, because it is impossible to generalize the approach to all languages, even to the languages of one language family, if they will have significant differences.

## Conclusion

Almost all languages in the Turkic family are low-resource languages. As these languages have words with similar roots and word formation rules, the proposed approach can help improve ASR models by providing multilingual training by combining datasets to train on the ESPNet system with CTC + Attention mechanism + LM. To maintain robustness, languages with similar letters (Cyrillic) were chosen for the study and experiment. The results showed that the ASR system chosen and the data combining approach can provide better results in comparison with models trained with data using mono language (Tables 6 and 7). This approach can also help solve the challenge of letter absence in critically low-resource languages. The proposed method can be applied to languages of other families, but different letters can lead to different results.

## Data Availability

The datasets analyzed during the current study are available by the link https://commonvoice.mozilla.org/en/datasets. Dataset was downloaded directly by web-browser. For each language was downloaded the version Common Voice Corpus 8.0. Dataset for multilingual corpus was formed by combining datasets of all included languages in the experiment to one big dataset.
